# Quantification of DNA through the NanoDrop Spectrophotometer: Methodological Validation Using Standard Reference Material and Sprague Dawley Rat and Human DNA

**DOI:** 10.1155/2020/8896738

**Published:** 2020-11-29

**Authors:** Alejandro Monserrat García-Alegría, Iván Anduro-Corona, Cinthia Jhovanna Pérez-Martínez, María Alba Guadalupe Corella-Madueño, María Lucila Rascón-Durán, Humberto Astiazaran-Garcia

**Affiliations:** ^1^Universidad de Sonora, Departamento de Ciencias Químico Biológicas, Hermosillo, Sonora CP 83000, Mexico; ^2^Centro de Investigación en Alimentación y Desarrollo, A.C. (CIAD AC), Coordinación de Nutrición, Hermosillo, Sonora CP 83304, Mexico

## Abstract

This study aimed to validate an analytical method to determine DNA concentration using standard reference material (NIST SRM 2372) and Sprague Dawley rat and human DNA. Microvolumes were used to analyse DNA samples. Linearity showed correlation coefficients higher than *R* ≥ 0.9950, and the precision value was ≤2% CV. Trueness based on bias and the percentage of recovery showed bias values lower than *Z*-test with a 95% confidence level and a recovery percentage within the range (% Rec = 100% ± 5%), and the stability of the samples was 60 days (2–4°C).

## 1. Introduction

Diagnostic service laboratories have been experiencing an increasing demand of molecular analysis; therefore, the implementation of good laboratory practices and quality assurance has become necessary. According to the Eurachem Guide, “The laboratory should use the test and calibration methods, including sampling, that satisfy the clients' necessities and that are appropriate for the assays being performed...” [1]. DNA analysis and quantification have become a common process in these laboratories daily as a starting point of the different procedures being performed in the molecular biology laboratory.

One of the most commonly used methods to estimate nucleic acid concentration is the measurement of sample absorbance at 260 nm [2]. The 260/280, 260/230, and 260/325 absorbance ratios are used to determine DNA purity and the presence of contaminants in the biological samples during the DNA extraction process [3, 4]. Currently, the most useful way to estimate DNA concentration and purity is through absorbance measures of samples' microvolumes using the NanoDrop spectrophotometer. Since its appearance [5], this normalised method has been used worldwide. Nevertheless, to the best of our knowledge, this methodology has not been integrally validated and the uncertainty of its measures has also not been determined, as recommended by international norms [1, 6]. That is, there are analytical parameters that must be determined to validate a measurement method. Among them are linearity, the limit of detection and quantification, precision under repeatability and reproducibility conditions, truthfulness through bias, and the recovery percentage and stability, among others. Recently, several researchers worldwide have committed to the validation of analytical methodologies to evaluate the DNA extraction process from microorganisms [7], to quantify DNA fragment amplification [8], for real-time PCR applications [9–13], for DNA extraction from glial cells [14], to evaluate DNA methylation in the human genome by microarrays [15], to validate sequencing assays in molecular oncology [16, 17], for genotyping of *β*-thalassemia genes [18], or to assess DNA methylation by Epicomet-chip assay [19]. Taking this into account, the objective of this study was to validate the NanoDrop spectrophotometry analytical method for DNA quantification. Validation was performed using human DNA standard reference material (NIST SRM 2372), as well as DNA from Sprague Dawley rats and human donors.

## 2. Experimental

### 2.1. Study Materials

The Human DNA NIST SRM 2372 was used to validate the analytical method for DNA quantification using the NanoDrop spectrophotometer. The results were compared with the DNA obtained from Sprague Dawley rats and human donors.

### 2.2. Collection of Blood Samples from Human Donors and Sprague Dawley Rats

Human blood samples were obtained from eight clinically healthy women aged between 35 and 50 years. The project characteristics were explained to them before obtaining their informed consent, as required by the national and international ethic regulations [20, 21]. Then, we proceeded with peripheral blood extraction through intravenous puncture, which was performed using a Vacutainer system in heparinised tubes [22]. This project was approved by the Ethics Committee of the Centro de Investigación en Alimentación y Desarrollo, A.C. (CIAD A.C.) (Folio CE/007/2018. Date of approval: 09 May 2018).

Blood samples were also collected from eight female Sprague Dawley rats (weight, 200 ± 20 g) who were not under any treatment and were kept in standard vivarium conditions: 12 h light/dark cycles, 18°C–24°C temperature, 40%–70% humidity, and standard rat food (LabDiet, imported from PetFood, Mexico), which was provided *ad libitum*. Rats were anaesthetised in a halothane chamber, and peripheral blood was then collected with the Vacutainer system in heparinised tubes [22]. All procedures were performed according to the national and international regulations to avoid animal suffering [23, 24]. This project was evaluated and approved by the Research Ethics Committee of the Universidad de Sonora (Folio: CBI-UNISON 1/2015. Date of approval: 09 February 2015).

### 2.3. Extraction, Purification, and Quantification of Rat and Human DNA

Total DNA extraction was performed according to the Chomczynski and Sacchi [25] method, with the TRIzol reagent. Furthermore, 0.050 ± 0.008 g of human and rat peripheral blood were weighed, and for sample homogenisation, PolyTron PT 2100 (Equinlab) was used. The purity and concentration of the DNA obtained were determined through 260/280 nm absorbance measures [3] using the NanoDrop spectrophotometer 2000 (Thermo Scientific) [26].

### 2.4. Validation of the NanoDrop DNA Quantification Method

The evaluation parameters of the DNA measurements in microvolumes (1 *µ*L) of samples were as follows: working range (linearity), detection and quantification limit using a blank (DEPC-treated), precision under conditions of repeatability and reproducibility, trueness based on bias and recovery percentage, and measurement stability.

#### 2.4.1. Linearity (Working Range)

Linearity was assessed through calibration curves generated by serial dilution (1 : 3) of the Standard Reference Material DNA and rat and human DNA. For serial dilution, ultrapure DEPC-treated (diethyl pyrocarbonate), nuclease-free water was used [27]. Five DNA concentrations were used in each calibration curve, and three replicates of each curve were obtained. Concentration was calculated by measuring the absorbance of 1 *µ*L of the sample at 260/280 nm using the NanoDrop 2000 spectrophotometer. A graph for linearity was plotted using the mean values of absorbance and the concentration of each point, and Pearson's correlation coefficient (*R*) and equation of the line were obtained. These parameters were obtained by plotting concentration against absorbance values using Excel 2010 [28].

#### 2.4.2. Limit of Detection (LOD) and Limit of Quantification (LOQ)

LOD and LOQ were evaluated using ultrapure DEPC-treated nuclease and phosphate-free water as a blank sample because it was the diluent used in all tests. In addition, 1 *µ*L of the blank was measured 30 times (*n*), and from these values, standard deviation (*sd*) and corrected standard deviation (*sd*´) were obtained. The corrected standard deviation is the standard deviation divided by$\sqrt{n}\;$, which is the number of blank readings obtained. Therefore, the equations to calculate LOD and LOQ are as follows:

(1) $$\begin{array}{c} \text{LOD}=3\text{ sd}{}^{\prime }, \end{array}$$

(2) $$\begin{array}{c} \text{sd}{}^{\prime }=\frac{\text{sd}}{\sqrt{n}}, \end{array}$$

(3) $$\begin{array}{c} \text{LOQ}=10\text{ sd}{}^{\prime }\text{.} \end{array}$$

#### 2.4.3. Precision under Repeatability and Reproducibility Conditions

Repeatability (*r*) was determined in the molecular biology laboratory of Patología Experimental de la Coordinación de Nutrición del CIAD. Reproducibility (*R*) was analysed in the Laboratorio de Investigación en Cáncer, Departamento de Ciencias Químico Biológicas of the Universidad de Sonora. Both parameters were determined using a NanoDrop 2000 spectrophotometer (Thermo Scientific). Three different DNA concentrations of NIST SRM 2372 and human and Sprague Dawley rat samples were prepared. The concentrations used were as follows: 23.6, 7.6, and 2.3 ng/*µ*L of SRM DNA and 30.0, 10.0, and 3.3 ng/*µ*L of rat and human DNA. Each DNA concentration was measured 30 times. Repeatability (*r*) and reproducibility (*R*) were estimated using the percentage of the coefficient of variation (% CV_*r*_ and % CV_*R*_) using the following equations:

(4) $$\begin{array}{c} \%{\text{CV}}_{r}=\frac{\text{sd}}{\bar{y}}*100,\\ \%{\text{CV}}_{R}=\frac{\text{sd}}{\bar{y}}*100. \end{array}$$

In this equation, sd is the standard deviation and $\bar{y}$ is the mean value of the 30 replicates. The acceptance criterion for instrumental methods is % CV ≤ 2 [1].

Another method to estimate repeatability (*r*) and reproducibility (*R*) is to calculate the variance to evaluate the precision of the repeatability (*sr*) and reproducibility (*sR*). The equation to calculate the variance (*S*^*2*^) is described as follows:

(5) $$\begin{array}{c} S^{2}=\sum \frac{{\left(xi-\bar{y}\right)}^{2}}{n-1}. \end{array}$$

In this equation, *xi* is the value of the individual measure, $\bar{y}$ represents the mean, and *n* is the size of the sample or the number of repetitions.

#### 2.4.4. Trueness

*(1) Based on Bias*. Trueness based on bias was determined using equations (6) and (7), for which three DNA concentrations were prepared: 23.6, 7.6, and 2.3 ng/*µ*L of NIST SRM 2372 DNA and 30.0, 10.0, and 3.3 ng/*µ*L of rat and human DNA. These concentrations represent the theoretical value (*μ*), and each of them was measured 30 times using the NanoDrop 2000 spectrophotometer. From the data obtained, the mean of each concentration was calculated (*ӯ*). For statistical analysis, a *Z*-test of distribution with a 95% confidence level (significance level of 5%, *α* = 0.05) was performed. The *sr* value is the precision error (typical standard deviation) under repeatability and reproducibility conditions of the method being evaluated. The 1.5 and 0.1 values are constants, and *n* is the number of measurements (*n* = 30).

(6) $$\begin{array}{c} \left[\bar{y}-\mu \right]\leq \frac{Z_{95\%}\cdot \text{sr}}{\sqrt{n}}, \end{array}$$

(7) $$\begin{array}{c} \text{sr}=1.5+\left(0.1*\bar{y}\right). \end{array}$$

If $\left[\bar{y}-\mu \right]\leq \left(Z_{95\%}\cdot sr\right)/\sqrt{n}$, then bias is equal to zero, and therefore, there is trueness in each level of measurement. In other words, the method is true or accurate [1].

*(2) Based on the Percentage of Recovery*. The percentage of recovery (% Rec) was obtained from the three previously selected concentrations: 23.6, 7.6, and 2.3 ng/*µ*L of NIST SRM 2372 DNA and 30.0, 10.0, and 3.3 ng/*µ*L of rat and human DNA. This parameter was evaluated as follows:

(8) $$\begin{array}{c} \%\text{Rec}=\frac{\bar{y}}{\mu }\times 100. \end{array}$$

The measured value (true) represents the mean ($\bar{y}$) of the concentration of each selected level for the 30 replicates, whereas *μ* is the theoretical value of each concentration. For SRM, the acceptance criteria established that the result should be % Rec = 100% ± 5% (95%–105% confidence interval), whereas the acceptance criterion for a standard or high purity sample is 100% ± 15% (85%–115% confidence interval) [1].

#### 2.4.5. Stability

To evaluate the stability of the DNA quantification method using the NanoDrop 2000 spectrophotometer, 7.6 ng/*µ*L NIST SRM 2372 DNA concentration was selected. To this end, 35 *µ*L DNA solution at a concentration of 7.6 ng/*µ*L was prepared and the DNA concentration was measured in 1 *µ*L of the solution every 2 days for 60 days (*n* = 30). For standard error determination, the following equation was used:

(9) $$\begin{array}{c} \text{Error}=yi-\mu . \end{array}$$

In this equation, *yi* is the individual measure and *µ* is the mean of the measurements. The error (±1 standard deviation) was plotted against time (days).

## 3. Results and Discussion

The process to validate the method was implemented based on the theory of errors [1]. Microvolume quantification systems remarkably reduce sample consumption and radically increase the concentration range when compared with more traditional quantification systems. This methodology has become a broadly accepted alternative to the traditional methods of nucleic acid quantification, even when the sample may be liberal [3]. Some researchers recommend quantifying DNA and/or RNA with methods different from the NanoDrop UV/Vis spectrophotometric determination because they argue that various molecules present in human serum samples, such as proteins and carbohydrates, can be contaminated, which interferes with the nucleic acid quantification [29, 30]. They recommend the usage of fluorometric techniques. However, the cost of fluorometric techniques is higher than that of the NanoDrop spectrophotometric method, thereby raising the cost of analysis. [2]. Taking into account the cost and time of analysis, volume of sample, and reliability of results, the NanoDrop spectrophotometric method is acceptable and advisable [31, 32]. Satisfactory results have been reported for the quantification of oral epithelia DNA using the NanoDrop 2000 spectrophotometer. Moreover, Price et al. [33] have designed an analytical strategy, similar to the one employed in this study, to validate the method for evaluation of *Burkholderia pseudomallei,* the causative agent of melioidiosis, through real-time PCR for clinical, environmental, and forensic assays. They evaluated accuracy, precision, LOD, LOQ, linearity, and selectivity among others with adequate results. In this sense, although several variants of PCR and molecules used in the validation process, such as miRNA, have been designed, it is important to assess the preanalytical conditions to achieve reliability in the results obtained [34]. Moreover, as Vigneron et al. [35] have pointed out, it is key to normalise the method to evaluate miRNA prior to cancer clinical tests.

### 3.1. Linearity (Working Range)

**Figure 1:**
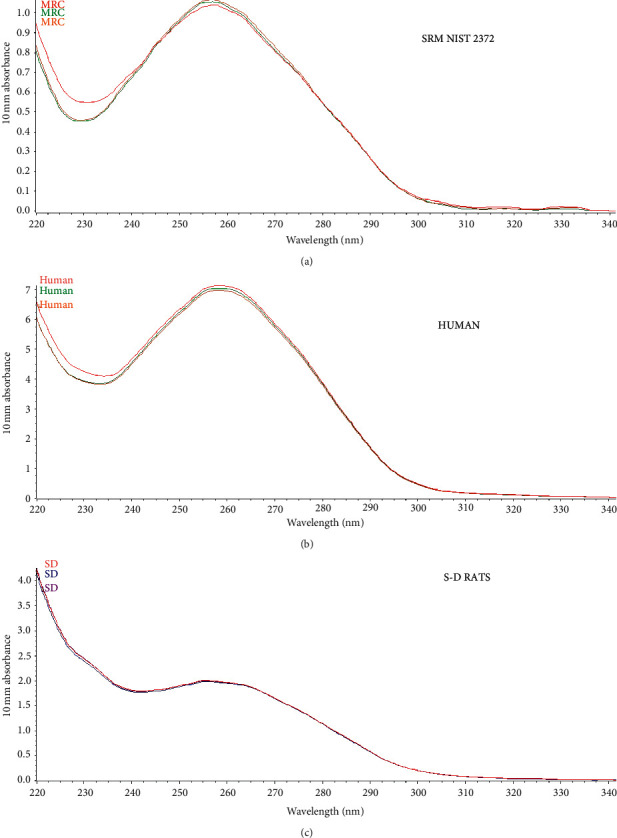
Spectroscopic characteristics of the DNA of (a) the certified reference standard (NISTSRM2372), (b) humans, and (c) Sprague Dawley rats.

Before the evaluation of the calibration curves, we verified that the UV spectra of the DNA samples from humans and Sprague Dawley rats were compatible with the UV spectra of DNA from the certified reference DNA (NIST SRM 2372). As can be seen in Figure 1, when comparing the absorbance spectra, the characteristic peak of the nitrogenous bases is at 260 nm and the valley at 230 nm characteristic of the proteins. The initial concentration of human DNA obtained from the peripheral blood was 91.55 ng/*µ*L, with a purity of 1.86; initial concentration of Sprague Dawley rat DNA was 57.9 ng/*µ*L, with a purity of 1.95; and the initial concentration of the NIST SRM 2372 was 57 ng/*µ*L quantified at 260 nm, with a purity of 2 determined at 260/280 nm. The DNA concentrations used in the calibration curves were 86.13, 28.10, 9.86, 3.20, and 1.20 ng/*µ*L for human DNA; 86.26, 29.13, 10.20, 3.73, and 1.26 ng/*µ*L for Sprague Dawley rat DNA, and 69.13, 23.73, 7.53, 2.06, and 0.23 ng/*µ*L for NIST SRM 2372 DNA. Absorbance values in the graph represent the mean of three replicates of each of the DNA samples. Pearson's correlation coefficient (*R*) was 0.9999, 1.0000, and 1.0000 for SRM, Sprague Dawley rats, and human DNA, respectively. Taking into account the acceptance criteria of *R* ≥ 0.9950, the calibration curves of the three DNA sources are acceptable according to [5].

Previous studies evaluating linearity through Pearson's correlation coefficient have obtained an *R* ≥ 0.9999 [36]. Some researchers have validated their methodologies for DNA microarrays and have obtained Pearson's correlation coefficients similar to those obtained in our study (*R* ≥ 0.994) [15, 37]. Dietrich et al. [17] evaluated the DNA methylation degree using real-time PCR and found an association between the degree of methylation of a specific gene (*PITX*) and real-time PCR (*R* = 0.9871); these findings show predictive values in assessing the recurrence of prostate-specific antigen levels in patients with prostate cancer. In addition, the method has been validated to evaluate the DNA methylation degree in a modified comet assay (Epicomet-chip); it has been observed that the methylation degree increases when the percentage of the comet tail is high (*R* = 0.7300), and the method was validated to quantify the concentration of the number of DNA copies by PCR using SRM [19, 38]. The linearity obtained between the number of measured copies and the expected number of copies of the SRM, based on Pearson's correlation coefficient, was *R* = 0.9985.

### 3.2. Limit of Detection (LOD) and Limit of Quantification (LOQ)

LOD and LOQ were determined using 30 measures of the blank (ultrapure DEPC water), determined at 260 nm wavelength using the NanoDrop 2000 spectrophotometer. The values were applied to equations (1)–(3), and the following LOD and LOQ results were obtained:

(10) $$\begin{array}{c} \text{sd}{}^{\prime }=\frac{s\;d}{\sqrt{n}}=\frac{0.4935}{\sqrt{30}}=0.0901,\\ \text{LOD}=3\text{ sd}{}^{\prime }=3\left(0.0901\right)=0.2703\;\text{ng}/µ\text{L},\\ \text{LOQ}=10\text{ sd}{}^{\prime }=10\left(0.0901\right)=0.901\;\text{ng}/µ\text{L}. \end{array}$$

The limit of detection (LOD) and the limit of quantification (LOQ) were determined according to what is established in the international guide Eurachem 2014 [1]. In this guide, it is established that both parameters can or should be estimated through the blank readings, which must correspond to the type of diluent used to make the dilutions that are made to obtain the calibration curves. This is corroborated in the NanoDrop 2000 equipment user manual [26]. Formerly, the way to calculate the LOD is by means of an equation that has become obsolete and that Eurachem itself changed in its last update [1] and taken from the equation of Miller and Miller, 2002 [39].

This equation works with blank readings, but takes into account the average of the readings and adds the standard deviation of the blanks to it. The equation is as follows:

(11) $$\begin{array}{c} \text{LOD}={\bar{y}}_{B}+3*\text{sd}=1.57\;\text{ng}/µ\text{L}+3*0.4935\;\text{ng}/µ\text{L}=3.05\;\text{ng}/µ\text{L}. \end{array}$$

If we consider the value of *n* (number of repetitions), the equation would be as follows:

(12) $$\begin{array}{c} \text{LOD}={\bar{y}}_{B}+3*\text{sd}/\sqrt{n}=1.57+3*0.4935/\sqrt{30}=1.84\;\text{ng}/µ\text{L}. \end{array}$$

This last value is very similar to value of 2.0 ng/*µ*L of the limit of detection (LOD) established by the supplier of the NanoDrop 2000 equipment. Finally, it should be clarified that, in the NanoDrop equipment user manual, the methodology used to estimate the LOD equivalent to 2 ng/*µ*L of DNA is not described [26].

In a study on validating a method to quantify forensic DNA for its use in real-time PCR, Spas and Zbieć-Piekarska [36] determined that LOQ was 11.5 pg/*µ*L of DNA using a human DNA quantification kit (Quantifiler). This value is much lower than that obtained in our study.

In addition, Forootan et al. [40] used a method to calculate LOD and LOQ based on the coefficient of variation that they obtained from the RT-qPCR calibration curve generated from human DNA.

### 3.3. Precision under Repeatability and Reproducibility Conditions

To evaluate precision under repeatability and reproducibility conditions, % CV was calculated.  Table 1 shows the results of both conditions, with three concentrations of each type of the sample.

The results obtained for repeatability and reproducibility that were evaluated using % CV are acceptable because the acceptance criterion for instrumental methods is % CV ≤ 2.0% [5], with the exception of the lower concentrations (2.71, 3.33, and 3.33 ng/*µ*L). At these lower concentrations, the repeatability and reproducibility of the measures could not be confirmed because these values are too close to the LOQ value previously calculated (LOQ = 0.901 ng/*µ*L).

However, this result is to be expected as the lower LOD reported for double-stranded DNA using the NanoDrop spectrophotometer is 2 ng/*µ*L (Thermo Scientific, 2009) [26]. In the NanoDrop equipment user manual, they used reproducibility values of ±2%, but do not describe what methodology they used to obtain this value as a cutoff or acceptance point [26]. Furthermore, it has been observed that the reproducibility decreases as the DNA concentration decreases below 17.5 ng/*µ*L. In contrast, the DNA LOQ for microbial DNA was approximately 11 ng/*µ*L [7]. Purity and DNA concentration were determined through UV spectroscopy. Regarding the low DNA concentration and its low recovery and repeatability, it has been observed for the determination of RNA concentration that when working with low concentrations of RNA, the results have low repeatability (Aranda et al., 2009). Spas and Zbieć-Piekarska [36] found that when DNA concentration decreases, the same occurs with the precision, evaluated with % CV. In other words, with DNA concentrations below 0.1 ng/*µ*L, the inaccuracy increases from 20% to 41%, indicating that the repeatability and reproducibility of the measures decrease. It is important to highlight that these studies accepted % CV ≤ 20%.

Moreover, the validation of the method for genetic mapping of DNA fragments using next-generation sequencing has obtained results of 98.6% bases mapped with a standard deviation (error) of 1.5%. These results are acceptable for other ways to validate analytical methods for oncologic material [41]. In this sense, reproducibility evaluations between analysts have been performed with acceptable results, for example, to assess mutations within single nucleotide variations; when five cancer variants were studied by next-generation sequencing techniques, the intra- and interanalyst values obtained were 96.3% and 98.1%, respectively [11]. Xiong et al. found 100% reproducibility for the Tm analysis (melting temperature) based on PCR assays for genotyping one of the *β*-thalassemia stages [18].

Ebentier et al. have estimated that, for the method to determine microbial DNA by PCR, the variation percentage of repeatability and reproducibility was 0.1%–3.3% and 1.9%–7.1%, respectively [42].

Finally, it is important to point out that the validation process uses analytical parameters, including linearity, detection limit, and precision under repeatability or reproducibility conditions, as well as truthfulness through bias and the recovery percentage and stability, among others, regardless of the analytical method used to make measurements with DNA. Sometimes, due to a lack of elements that allow a more objective comparison with the results obtained by other researchers who have worked with the same method, some of these parameters are indirectly compared.

### 3.4. Trueness

#### 3.4.1. Based on Repeatability and Reproducibility Bias

Trueness based on bias was evaluated for repeatability (*S*_*r*_) and reproducibility (*S*_*R*_), for which the variation coefficient percentage was calculated.  Table 2 shows the results for both conditions, with three concentrations for each type of the sample. For all the samples and DNA concentrations, the bias was lower than the statistical test used (*Z*-test) with a 95% confidence level. Therefore, the bias is not significant; hence, trueness of the measurements exists [1].

#### 3.4.2. Based on the Percentage of Repeatability, Reproducibility, and Recovery

Trueness based on bias was evaluated based on the percentage of repeatability, reproducibility, and recovery, for which % CV was calculated.  Table 2 shows the results of both conditions, with three concentrations for each type of sample. The results indicate that the recovery percentage for the NIST SRM 2372 DNA was within the acceptability range (% Rec = 100% ± 5%) [1]. The % Rec for Sprague Dawley rats and human DNA were also within the range (% Rec = 100% ± 15%) [1].

### 3.5. Stability

**Figure 2:**
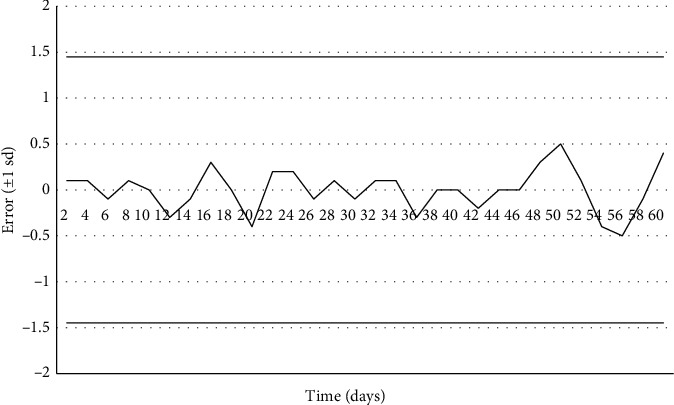
Stability of DNA dissolution from NIST SRM 2372.

As can be seen in Figure 2, when the error was plotted with a standard deviation of ±1 (±1 sd) against time (days), none of the measurements were located outside the lower or higher interval, which indicates that the solution used is stable for at least 60 days, when kept at a refrigeration temperature of 2°C–4°C.

It is important to consider the validation process of a method for quantifying or selecting candidates or endogenous control genes (housekeeping) for RT-qPCR (RQ-PCR) because some of them overcome changes in their expression under different pathological states or certain pharmacological treatments [10]. This is especially relevant for evaluating the stability of expression of endogenous control genes (housekeeping) [13]. Finally, He et al. [43] pointed out the importance of determining the method with which genomic DNA concentration will be evaluated because the results obtained using different methods differ. For all of the results conveyed above, it is of great importance to validate analytical methods.

Our findings confirm the parameters offered by the NanoDrop operating manual. Additionally, validation was performed through the Error Theory [1], in which analytical parameters such as linearity, detection and quantification limit, precision under conditions of repeatability and reproducibility, and truthfulness through bias and recovery percentage, as well as stability, were evaluated as recently established by international regulations [1]. Our results allow recommending the quantification method for DNA concentrations above 3.5 ng/*µ*L. When experiments require greater sensitivity, we recommend using a fluorimeter for low concentrations of DNA. For high precision applications, we recommend using automated microfluidic-based electrophoresis.

On the other hand, a measurement method can also be validated through the estimation of expanded uncertainty, using the Law of Propagation of Uncertainty [44], and that is something we are working on for publication soon.

Finally, our research group has recently worked with genetic material from different experimental animals, and based on these works, we decided to develop a research project that would allow validating the measurement method, first through the Theory of Errors and then through the Estimation of Expanded Uncertainty, and these results can be used as analytical indicators by other researchers.

## 4. Conclusions

According to the international regulations, methodological validation represents the most important tool to determine if an analytical method is adequate for a particular purpose and, thus, be able to guarantee that the results of a measurement are reliable. For this purpose, it is essential to use SRMs that guarantee the reliability of the results and then to compare it against the same type of material but of a different origin. Hence, it is necessary to count the cutoff points from which it can be established if an analytical parameter is reliable or if it is within the acceptability ranges. The linearity results, evaluated based on the correlation coefficients, were higher than the acceptance criteria (*R* ≥ 0.9950) and, therefore, are reliable. The results obtained for the precision parameter under repeatability and reproducibility conditions are also reliable because their coefficient of variation is lower than 2% (acceptance criteria for instrumental methods CV ≤ 2%), with the exception of DNA concentrations below 3.5 ng/*µ*L, where the precision results are over 2% CV. The results obtained from the trueness based on bias (*s*) and recovery percentage (% Rec) are reliable because in the three concentrations assessed, the bias was lower than the statistical test (*Z*-test) with a 95% confidence interval. Besides, the recovery percentage for the NIST SRM 2372 DNA was between 98% and 102% (acceptance criteria % Rec = 100% ± 5%) and between 85% and 115% for Sprague Dawley rats and human DNA (acceptance criteria % Rec = 100% ± 15%). Lastly, although the results obtained for the stability of the samples are good, they are not considered a quality parameter in the international quality guidelines for measurement methods and show a stability of at least 60 days, when kept at a refrigeration temperature of 2°C–4°C, with a standard deviation between the ±1 range. In conclusion, the validation process of the DNA quantification method by NanoDrop spectrophotometry using NIST SRM 2372 DNA, as well as Sprague Dawley rat and human DNA, has proved that the method is safe and reliable. Therefore, its implementation in research or service laboratories is feasible and safe.

## Figures and Tables

**Table 1:** Repeatability and reproducibility assessment for three DNA concentrations.

	DNA concentration (ng/*µ*L)								
	NIST SRM 2372			Sprague Dawley rats			Humans		
	23.60	7.61	2.71	30.00	10.00	3.33	30.00	10.00	3.33
% CV_r_	1.12	1.78	13.15	1.25	2.02	7.27	1.13	1.91	8.79
% CV_R_	0.89	1.73	6.16	1.18	1.71	8.66	0.85	1.89	9.56

% CV_r_ = Percentage of repeatability coefficient of variation. %CV_R_ = Percentage of reproducibility coefficient of variation.

**Table 2:** Assessment of truthfulness from bias for repeatability and reproducibility and from percent recovery for three DNA concentrations.

	DNA concentration (ng/*μ*L)								
	NIST SRM 2372			Sprague Dawley rats			Humans		
	23.60	7.61	2.71	30.00	10.00	3.33	30.00	10.00	3.33
S_r_	0.053	0.006	0.050	0.070	0.560	−0.350	−1.426	−0.360	−0.486
S_R_	−0.093	−0.076	−0.073	0.160	0.170	0.256	−0.113	−0.156	−0.190
% Rec_r_	100.22	100.08	101.85	100.23	105.63	89.39	95.24	96.40	85.25
% Rec_R_	98.55	98.99	102.60	100.51	101.65	107.52	99.62	98.43	94.24

S_r_ _=_ repeatabiliy bias_._ S_R_ _=_ reproducibility bias. % Rec_r_ _=_ repeatability recovery percentage. % Rec_R_ _=_ reproducibility recovery percentage.

## Data Availability

The original data used to support the findings of this study are available from the corresponding author upon request.
